# Danish Population Based Study of Familial Epilepsy and Childhood Cancer

**DOI:** 10.21203/rs.3.rs-2500755/v1

**Published:** 2023-04-14

**Authors:** Corbin Platamone, Xiwen Huang, Rajarshi Mazumder, Beate Ritz, Jorn Olsen, Johnni Hansen, Chai Saechao, Julia E Heck

**Affiliations:** UCLA School of Public Health: University of California Los Angeles Jonathan and Karin Fielding School of Public Health; UCLA School of Public Health: University of California Los Angeles Jonathan and Karin Fielding School of Public Health; David Geffen School of Medicine: University of California Los Angeles David Geffen School of Medicine; UCLA School of Public Health: University of California Los Angeles Jonathan and Karin Fielding School of Public Health; Aarhus University Department of Clinical Medicine: Aarhus Universitet Institut for Klinisk Medicin; Danish Cancer Society: Kraeftens Bekaempelse; UCLA Health System: University of California Los Angeles Health System; UCLA School of Public Health: University of California Los Angeles Jonathan and Karin Fielding School of Public Health

**Keywords:** In-utero exposure, Danish population based study, Maternal Epilepsy, Childhood cancer, Antiepileptic medications

## Abstract

**Objective:**

Results from studies investigating the association between maternal or child epilepsy, use of anticonvulsants in pregnancy, and childhood cancer are inconsistent and at times contradictory.

**Methods:**

Linking Danish national databases, we obtained epilepsy and childhood cancer diagnoses, and anticonvulsant use data. We estimated adjusted odds ratios of all or specific childhood cancers in relation to maternal or child epilepsy and anticonvulsant therapies using conditional logistic regression.

**Results:**

Maternal epilepsy was positively associated with all childhood cancers in offspring, specifically, with acute lymphoblastic leukemia (Odds Ratio (OR) = 1.68, 95% Confidence Interval (CI) = 1.16, 2.43) and Wilms tumor (OR = 2.13, 95%CI = 0.97, 4.68). When considering maternal ever (lifetime) ingestion of anticonvulsants, a positive association was found with all cancers (OR = 1.15, 95%CI = 1.01, 1.31), and central nervous system tumors (OR = 1.32, 95%CI = 1.03, 1.69) as well as neuroblastoma (OR = 2.05, 95%CI = 1.29, 3.28) among offspring. Maternal anticonvulsant use before or during the index pregnancy was related to CNS tumors in offspring (OR = 1.78, 95%CI = 0.99, 3.21), however the confidence interval included the null.

**Significance:**

Maternal use of certain anticonvulsant medications may be a risk factor for cancer in offspring. Medical providers may need to consider what type of treatments to prescribe to pregnant mothers with epilepsy.

## Introduction

Approximately 400,000 children between the ages of 0–14 develop cancer globally each year [1, 2]. The most common types of childhood cancers are leukemias, neuroblastomas, brain tumors, and Wilms’ tumors. [3] Even though this only accounts for less than 1 percent of the total annual global cancer incidence, [4] cancer remains the second leading cause of childhood morbidity in developed countries in this age group. [5] Suspected causes of childhood cancer include medication use, agricultural or industrial chemicals, and tobacco smoke (and hepatoblastoma) in pregnancy as well as hereditary and acquired mutations. [6-9]

Worldwide approximately 7.2 million women of childbearing age have epilepsy. [10] One study found that approximately 70 percent of women with epilepsy reported adhering to their prescribed epilepsy medications during pregnancy. [11] Yet, some reports found that 40–60 percent of pregnant women with epilepsy reported having been non-compliant with their medications. [12, 13] Mothers with epilepsy are slightly more likely to undergo Cesarean section, deliver preterm, and have offspring with low birth weight even when not using antiepileptic drugs. [14, 15] Anticonvulsants can be transmitted to the fetus transplacentally, [16] and research has established strong teratogenic effects from pregnancy exposure. [14, 17-19] In addition, adults with epilepsy may be at an increased cancer risk. A recent cohort study in Denmark found moderate increases in total cancer incidence and specifically increases in liver, mouth and throat, central nervous system, and respiratory cancers among adults with epilepsy who took anticonvulsants. [20] Yet, despite the evidence for both gestational teratogenic and adult carcinogenic impacts, there have been relatively few studies evaluating whether offspring of parents with epilepsy are at an increased cancer risk, likely due to the relative rarity of both parental epilepsy and cancer in children. [21, 22]

Previous studies have suggested a relationship between particular types of anticonvulsants, such as barbiturates and phenytoin, and the development of various cancers in animal models. [23, 24] With regards to childhood cancer, elevated risks were reported for acute lymphoblastic lymphoma (ALL) with non-specific maternal intake of anti-epileptic medications in pregnancy [25] as well as for brain tumors, although some studies had small sample sizes and reported wide confidence intervals that included the null. [26-28] A link between childhood lymphoma and prior exposure to phenytoin in utero has also been proposed [29], but several studies were unable to corroborate these associations. [30-33] A study that found an association with brain tumors in children taking anticonvulsants or having a history of epileptic seizures, may have been affected by reverse causation if the tumor was causing seizures prior to diagnosis. [33]

This study attempts to determine whether children are at a higher risk of developing cancers from exposure to anticonvulsant medications in utero. We also evaluated the possibility that the mother’s epilepsy plays an independent etiologic role in the development of cancer in their offspring.

## Materials And Methods

This study was conducted in Denmark via a linkage of large national databases and has been described in detail previously [34]. Epilepsy diagnosis (Supplemental Table 1) was based on the International Classification of Diseases (ICD-O) until 2003 and the International Classification of Diseases, Revision 10 (ICD-10) thereafter, and were ascertained from the National Patient Registry. Childhood cancer cases (0–19 years old) were retrieved from the Danish Cancer Registry, which contains information on the morphology, topography, and date of diagnosis, among other factors. The subtype of cancer was based on morphology recorded in the International Classification of Childhood Cancer (ICCC) revision one until 2003 and revision three thereafter. For central nervous system tumors, we included both malignant and benign tumors. Although astrocytomas are a subtype of gliomas and the cases may overlap, we reported results for both in order to compare to previous studies. [26, 33]

We additionally linked our data to the Medical Births Register for information on gestational characteristics, and to the Central Population Register that links parents to their children and provides demographic information. From the Central Population Register we randomly selected controls that we matched to cases by sex and birth date (25:1 matching rate). In order to use information from registries on pregnancy, we restricted the study to children whose mothers were pregnant and gave birth while in Denmark.

Although our primary interest was to examine cancer risks in children in relation to maternal epilepsy or anticonvulsant use during pregnancy, we considered that epilepsy and childhood cancer may have shared genetic risk factors and thus also needed to account for paternal history of epilepsy. [35] We therefore additionally examined childhood cancer in relation to paternal epilepsy.

Due to differences in data availability for diseases and treatments, we generated two analytical datasets (Supplemental Chart 1):

Dataset #1 for births in 1977–2013 and cancer diagnoses in 1977–2016 i.e., from the first establishment of the National Patient Registry in 1977 onwards. These analyses included 6,420 cases and 160,484 controls. Pregnancies that were likely not viable (gestational age < = 20 weeks or birth weight < 500g, n = 17) were excluded from these analyses.

Dataset #2 is based on the pharmaceutical registry and births in 1995–2014 and cancer diagnoses in 1995–2016 where we linked study subjects to the National Pharmaceutical Registry (established in 1996 with pilot exposure data collected and available beginning in 1995), which provides information on every prescription filled at a pharmacy in Denmark. For the analysis based on the second dataset, we ascertained epilepsy diagnoses from the National Patient Register and additionally via identification of individuals prescribed medications indicated solely for epilepsy (Supplemental Table 1). The National Pharmaceutical Registry lists the dates of all medications filled, and information on medication and dose, using Anatomical Therapeutic Chemical Classification (ATC) codes. As this sample was created later, matched controls were also re-sampled from the Danish population. These analyses included 2,521 cases and 63,014 controls. Pregnancies likely not viable (gestational age < = 20 weeks or birth weight < 500g, n = 11) were excluded from analyses.

### Statistical Analyses:

We estimated the crude odds ratios of cancer diagnosed in the offspring using conditional logistic regression to account for matching and report results for all cancer types with at least five exposed cases. Potential confounding variables considered for adjustment were suggested by the literature [9, 32, 36-39]. In our final model, we adjusted for maternal age (continuous), maternal history of migraines (ever/never), and child’s epilepsy before cancer diagnosis (ever/never). We considered additional adjustment for urbanicity of residence, maternal smoking, maternal diabetes, familial socioeconomic status, and maternal asthma but did not include these factors in the final regression model because they did not change the estimates by 10% or more. [40] Earlier studies suggested an increased risk of lymphoma in mice exposed to phenytoin [24] and brain tumors in children whose mothers used barbiturates during pregnancy [41]. We had sufficient statistical power to examine childhood cancer risk for barbiturates but not phenytoin.

Since the mechanisms and presenting symptoms as well as treatment for epileptic seizures vary by the epileptogenic regions of the brain, [42] we separately examined the risk of cancer in offspring of mothers diagnosed with primary generalized epilepsy and focal epilepsy. Epilepsy subtypes were identified using ICD codes listed in Christensen [43].

Seizures may be a presenting symptom of various brain tumors, [39] thus our analyses of children’s epilepsy in relation to cancer may be at risk of reverse causality. We therefore did not examine childhood epilepsy in relation to cancer diagnosis.

All statistical analyses were conducted using SAS, Version 9.4 (SAS Institute Inc., Cary, NC, USA). Human subject approvals were obtained from the University of California Los Angeles, and the Danish Data Protection Board.

## Results

### Dataset #1

We present demographic and health data for the study population in Supplemental Table 2. Compared to mothers without epilepsy, mothers with epilepsy tended to be younger and were more likely to live in rural areas. A larger proportion of those who were diagnosed with epilepsy reported having migraines, diabetes, asthma, and smoked at the first prenatal visit compared to those who did not have epilepsy.

Table 1 presents the association between childhood cancer diagnoses in relation to maternal epilepsy diagnosis. Maternal epilepsy was positively associated with all cancers as well as acute lymphoblastic leukemia (ALL) and Wilms tumor.

When considering maternal epilepsy subtypes (Supplemental Table 3), increases in risk were seen with focal epilepsy and ALL (Odds Ratio (OR) = 2.39, 95% Confidence Interval (CI) = 1.15–4.96) and gliomas (OR = 3.05, 1.37–6.81). Increased risk for astrocytoma was seen with generalized epilepsy (OR = 2.99, 95% CI 1.09–8.97). We did not find any association between paternal epilepsy and childhood cancers (Supplemental Table 4).

### Dataset #2

Compared to mothers without epilepsy, the majority of mothers diagnosed with epilepsy in this subsample were younger at the time of the offspring’s birth while the largest proportion of mothers without epilepsy were between 30–34 years of age at that time (Supplemental Table 5). A higher proportion of this subgroup of mothers with epilepsy also resided in rural areas, had migraines, diabetes, and asthma prior to pregnancy compared to mothers without epilepsy.

Table 2 presents the association between maternal anti-epileptic medication use and childhood cancer. When considering maternal ever (lifetime) use of anticonvulsants, a positive association was found with all cancers (OR = 1.15, 95%CI = 1.01, 1.31), and central nervous system tumors (OR = 1.32, 95%CI = 1.03, 1.69) as well as neuroblastoma (OR = 2.05, 95%CI = 1.29, 3.28). When we examined maternal intake before or during the index pregnancy, a positive association was suggested for central nervous system tumors, but confidence intervals contained the null (OR = 1.78, 95%CI = 0.99, 3.21). Limiting to barbiturates, there were only 5 exposed childhood cancer cases, and the estimate was uninformative for all childhood cancers. (OR = 1.54, 95% CI 0.62, 3.80).

## Discussion

In this population-based study of childhood cancer, we found that maternal epilepsy –irrespective of anticonvulsant medication use – was positively associated with acute lymphoblastic leukemia and Wilms tumors in offspring. Additionally, maternal anticonvulsant use was linked to CNS tumors and neuroblastoma in offspring. In contrast, we did not observe associations with paternal epilepsy. The lack of association with paternal epilepsy would suggest that the increased risk of cancer is likely maternally influenced, either directly from maternal use of anticonvulsant medications, or indirectly via negative impacts of maternal epilepsy on pregnancy.

Such negative impacts on pregnancy have been the focus of some past studies targeting clinician guidelines. A recent systematic review and meta-analysis found increased risks of spontaneous miscarriage, ante and post-partum hemorrhage, caesarean section, hypertensive disorder, and induction of labor among mothers with epilepsy, primarily among those who took anticonvulsant medications [44]. Another study found elevated risks of anemia, uterine fibroids, ovarian cysts, and thyroid dysfunction among pregnant epileptic mothers [10]. Hypothyroidism has recently been linked to childhood cancer [45].

However, the degree to which the mother’s epileptic condition itself or the medications used may be responsible for developing childhood cancer can be difficult to elucidate. In our study, we cannot rule out that the association noted between maternal epilepsy and childhood cancer is due to confounding by indication. Previous literature has suggested that epileptic mothers, independent of anticonvulsant medications, are at a slightly higher risk of having low birthweight or preterm offspring (OR’s: 1.1-1.3), [14, 15] which has been linked to the development of childhood cancers such as gliomas and retinoblastomas [46, 47]. This seems to support that the mother’s condition related to adverse birth outcomes possibly contribute to the carcinogenic process regardless of medication use, but our results are inconclusive. When we examined women with epilepsy stratified by medication use, we observed lower point estimates among medication users compared to non-users and confidence intervals included the null (Supplemental Table 6). This may be due to the fact that only 7 case women with epilepsy were reported with a registered ATC code. This is contrary to previous reports indicating high adherence to medications prescribed during pregnancy. [48] Yet, our observations were comparable to another Danish population-based study which found similar rates of anticonvulsant consumption during pregnancy. [49] We were not able to conclude whether the observed increased risk of cancer in offspring is a result of maternal medication use or epilepsy diagnosis, as both estimates have wide and overlapping confidence intervals.

The teratogenic impacts of anticonvulsant therapies are well recognized. It has been established that some anticonvulsant medications used to treat epilepsy, particularly barbiturates and phenytoin, are potent carcinogens in animal models,[23, 24] and these drugs have been observed in human studies passing through the transplacental barrier to the fetus. [16] A study of anticonvulsant medications used in pregnancy found increased risks for neural tube defects, oral clefts, and heart defects among offspring, but conclusions were limited to valproic acid and carbamazepine consumed during the first trimester. [50] Another study found similar malformations, including spina bifida and trisomy 18, [51] in offspring exposed to anticonvulsants in utero, and yet another found minor craniofacial and digital malformations and concluded that the mother’s condition may have played a role. [52] Several of these birth defects are also related to childhood cancers. [53, 54]

We also considered examining the relationship between childhood epilepsy and cancers, and we observed elevated estimates for central nervous system tumors, including gliomas. This was expected as it is well known that seizures may be a presenting symptom of such brain tumors, [39] yet a 2-year lagged analysis still produced elevated estimates. This suggests multiple possibilities. Slow growing brain tumors may remain undiagnosed beyond our lagging cut-off, and therefore these seizures may in fact be a product of the cancer itself. [27] Conversely, the medications used to treat them could also be involved in the carcinogenic process. There are past studies that have found elevated cancer risks among children exposed to anticonvulsants, notably barbiturates, either in utero or in early childhood. [25-29, 33, 41] However, limited sample size made it impossible to conduct a lagged analysis beyond 2 years, and delays in tumor diagnosis would require far longer lags. [55] Seizures are also a common symptom of individuals with gliomas. [56] As such, we were unable to address the possibility of reverse causation.

We did not find any association for maternal barbiturate use. Our point estimate was lower than what has been seen in other studies, however our analysis was limited by the small number of exposed children (5 exposed cases and 80 exposed controls), likely due to decreasing use of barbiturates in high income countries. [57]

Our study design did not allow us to confirm that all mothers with a registered ATC code for an anticonvulsant purchase actually ingested the medication. In previous studies, noncompliance in pregnancy ranged from one-fifth to two-third for antiepileptic drug users; still others continued treatment but switched to safer alternatives. [14, 48, 58, 59] However, four-fifth of mothers with epilepsy in our study did not purchase anticonvulsant medications during pregnancy. Yet, noncompliance would have been non-differential as none of the mothers could have predicted the child’s cancer and it would have biased our results towards the null, thus the increased point estimates are notable.

Another factor to consider from our study is that some of these older drugs studied in the past, such as phenobarbital, may be facing increased prescription hesitancy from medical providers in high income countries. First generation anti-epileptic drugs were not subject to the same preclinical carcinogenicity testing required for drugs developed in the last thirty-eight years. [60] This shift towards more current antiepileptic medications may decrease the relevance of our study for those regions, however phenobarbital is still widely prescribed as a treatment for epilepsy in most of low-and middle-income countries, where 80% of people with epilepsy live. [61]

In summary, this study provides some indication that certain anticonvulsant medications may be carcinogenic to children exposed in utero. While our data does not seem to show an association between maternal epilepsy diagnosis when anticonvulsant medications are not used, maternal epilepsy has been associated with conditions linked to childhood cancer. Untreated epilepsy can have perinatal risks, thus discontinuation of anticonvulsant use may not be the safest option for patients. However, as certain anticonvulsant medications can irreparably harm the fetus, these conditions and treatments require consideration and further study.

## Figures and Tables

**Table 1:** Maternal Ever Diagnosis of Epilepsy in Relation to Childhood Cancer Risk, Dataset #1

Cancer Type	Total N	Exposed N (%)	Adjusted OR (95% CI) a*	Adjusted OR (95% CI) b*
**Controls**	160484	2534 (1.6)	Referent	Referent
**All Cancers**	6420	124 (1.9)	1.21 (1.01, 1.45)	1.16 (0.97, 1.40)
**Acute Lymphoblastic Leukemia**	1222	31 (2.5)	1.68 (1.16, 2.43)	1.68 (1.16, 2.43)
**Hodgkin lymphoma**	352	8 (2.3)	1.38 (0.66, 2.85)	1.36 (0.66, 2.82)
**Central Nervous System Tumors**	1583	35 (2.2)	1.37 (0.97, 1.94)	1.21 (0.84, 1.73)
**Astrocytomas**	502	11 (2.2)	1.34 (0.72, 2.49)	1.20 (0.63, 2.29)
**Intracranial and Intraspinal Embryonal Tumors**	674	13 (1.9)	1.09 (0.62, 1.91)	1.09 (0.62, 1.91)
**Gliomas**	778	19 (2.4)	1.46 (0.91, 2.34)	1.31 (0.80, 2.15)
**Neuroblastoma**	275	8 (2.9)	1.70 (0.82, 3.54)	1.64 (0.79, 3.44)
**Wilms Tumor**	203	7 (3.5)	2.12 (0.97, 4.67)	2.13 (0.97, 4.68)
**Germ Cell Tumors**	336	5 (1.5)	0.90 (0.36, 2.23)	0.88 (0.36, 2.19)
**Melanoma**	190	5 (2.6)	1.67 (0.66, 4.19)	1.60 (0.64, 4.04)

AOR values generated by simultaneous entry of covariates into a conditional logistic regression model

a*: Adjusted for maternal age and maternal history of migraine

b*: Adjusted for maternal age, maternal history of migraine, child's epilepsy before cancer diagnosis, and maternal history of cancer

**Table 2:** Maternal Antiepileptic Use in Relation to Childhood Cancer, Dataset #2

Cancer Type	N (%)	Crude OR (95% CI)	Adjusted OR (95% CI) *a
**Maternal Lifetime Use**			
Controls	5791 (9.2)	Referent	Referent
All Cancers	270 (10.7)	1.19 (1.04, 1.35)	1.15 (1.01, 1.31
Acute Lymphoblastic Leukemia	52 (9.3)	1.04 (0.78, 1.38)	1.06 (0.79, 1.41)
Acute Myeloid Leukemia	13 (11.4)	1.30 (0.73, 2.33)	1.27 (0.71, 2.29)
Hodgkin Lymphoma	15 (15.2)	1.49 (0.86, 2.58)	1.39 (0.80, 2.44)
Non-Hodgkin Lymphoma	12 (11.1)	1.23 (0.67, 2.25)	1.26 (0.69, 2.33)
Central Nervous System Tumors	78 (13.1)	1.47 (1.16, 1.87)	1.32 (1.03, 1.69)
Ependymoma	5 (12.2)	1.40 (0.55, 3.58)	1.26 (0.48, 3.26)
Astrocytoma	23 (13.9)	1.55 (0.99, 2.41)	1.34 (0.86, 2.12)
Intracranial and Intraspinal Embryonal Tumors	28 (12.7)	1.30 (0.87, 1.93)	1.28 (0.85, 1.91)
Gliomas	30 (12.7)	1.38 (0.94, 2.03)	1.21 (0.82, 1.79)
Neuroblastoma	22 (16.3)	2.13 (1.34, 3.37)	2.05 (1.29, 3.28)
Retinoblastoma	8 (11.8)	1.51 (0.72, 3.17)	1.59 (0.76, 3.36)
Unilateral Retinoblastoma	7 (15.6)	2.08 (0.92, 4.68)	2.18 (0.96, 4.95)
Rhabdomyosarcoma	5 (9.1)	1.00 (0.40, 2.51)	1.04 (0.41, 2.64)
Wilms Tumor	10 (10.4)	1.28 (0.66, 2.48)	1.27 (0.65, 2.48)
Bone Tumors	10 (11.5)	1.19 (0.61, 2.30)	1.11 (0.57, 2.17)
Ewing Sarcoma	6 (14.6)	1.60 (0.67, 3.82)	1.49 (0.62, 3.61)
Germ Cell Tumors	5 (5.5)	0.54 (0.22, 1.34)	0.51 (0.20, 1.27)

**Maternal Use Before or During Index Pregnancy**			
Controls	596 (1.0)	Referent	Referent
All Cancers	29 (1.2)	1.22 (0.84, 1.77)	1.13 (0.78, 1.65)
Central Nervous System Tumors	12 (2.0)	2.66 (0.65, 10.84)	1.78 (0.99, 3.21)
Acute Lymphoblastic Leukemia	7 (1.3)	1.30 (0.62, 2.76)	1.31 (0.62, 2.78)

**Maternal Use During Index Pregnancy**			
Controls	222 (0.4)	Referent	Referent
All Cancers	14 (0.6)	1.58 (0.92, 2.72)	1.47 (0.86, 2.54)
Central Nervous System Tumors	5 (0.8)	2.38 (0.98, 5.80)	1.99 (0.80, 4.93)

a*: Adjusted for maternal age, maternal history of migraine, and maternal history of cancer

## Data Availability

Restrictions apply to the availability of these data, which were used under license in this study. EU regulations govern the availability of Danish Data.

## References

[1] Ward Z.J., , Estimating the total incidence of global childhood cancer: a simulation-based analysis. Lancet Oncol, 2019. 20(4): p. 483–493. 30824204 10.1016/S1470-2045(18)30909-4

[2] Steliarova-Foucher E., , International incidence of childhood cancer, 2001-10: a population-based registry study. Lancet Oncol, 2017. 18(6): p. 719–731. 28410997 10.1016/S1470-2045(17)30186-9 PMC5461370

[3] Miller R.W., Young J.L. Jr., and Novakovic B., Childhood cancer. Cancer, 1995. 75(1 Suppl): p. 395–405. 8001010 10.1002/1097-0142(19950101)75:1+<395::aid-cncr2820751321>3.0.co;2-w

[4] Bhakta N., , Childhood cancer burden: a review of global estimates. Lancet Oncol, 2019. 20(1): p. e42–e53. 30614477 10.1016/S1470-2045(18)30761-7

[5] Kaatsch P., Epidemiology of childhood cancer. Cancer Treat Rev, 2010. 36(4): p. 277–85. 20231056 10.1016/j.ctrv.2010.02.003

[6] Zahm S.H. and Devesa S.S., Childhood cancer: overview of incidence trends and environmental carcinogens. Environ Health Perspect, 1995. 103 Suppl 6: p. 177–84. 10.1289/ehp.95103s6177 PMC1518893 8549470

[7] Savitz D.A. and Chen J.H., Parental occupation and childhood cancer: review of epidemiologic studies. Environ Health Perspect, 1990. 88: p. 325–37. 2272330 10.1289/ehp.9088325 PMC1568023

[8] Knudson A.G. Jr., Genetics and the etiology of childhood cancer. Pediatr Res, 1976. 10(5): p. 513–7. 180483 10.1203/00006450-197605000-00001

[9] Baldwin R.T. and Preston-Martin S., Epidemiology of brain tumors in childhood–a review. Toxicol Appl Pharmacol, 2004. 199(2): p. 118–31. 15313584 10.1016/j.taap.2003.12.029

[10] Thomas S.V., , Maternal and obstetric outcome of women with epilepsy. Seizure, 2009. 18(3): p. 163–6. 18805707 10.1016/j.seizure.2008.08.010

[11] Gurumurthy R., Chanda K., and Sarma G., An evaluation of factors affecting adherence to antiepileptic drugs in patients with epilepsy: a cross-sectional study. Singapore Med J, 2017. 58(2): p. 98–102. 26805666 10.11622/smedj.2016022 PMC5311891

[12] Lupattelli A., Spigset O., and Nordeng H., Adherence to medication for chronic disorders during pregnancy: results from a multinational study. Int J Clin Pharm, 2014. 36(1): p. 145–53. 24162929 10.1007/s11096-013-9864-y

[13] Fairgrieve S.D., , Population based, prospective study of the care of women with epilepsy in pregnancy. BMJ, 2000. 321(7262): p. 674–5. 10987772 10.1136/bmj.321.7262.674 PMC27482

[14] Veiby G., , Pregnancy delivery, and outcome for the child in maternal epilepsy. Epilepsia, 2009. 50(9): p. 2130–9. 19490036 10.1111/j.1528-1167.2009.02147.x

[15] Artama M., , Effects of maternal epilepsy and antiepileptic drug use during pregnancy on perinatal health in offspring: nationwide, retrospective cohort study in Finland. Drug Saf, 2013. 36(5): p. 359–69. 23615755 10.1007/s40264-013-0052-8

[16] Melchior J.C., O. Svensmark, and D. Trolle, *Placental transfer of phenobarbitone in epileptic women, and elimination in newborns*. Lancet, 1967. 2(7521): p. 860–1. 12387244 10.1016/s0140-6736(67)92593-7

[17] Tomson T., Battino D., and Perucca E., Teratogenicity of antiepileptic drugs. Curr Opin Neurol, 2019. 32(2): p. 246–252. 30664067 10.1097/WCO.0000000000000659

[18] Koch S., , Long-term neuropsychological consequences of maternal epilepsy and anticonvulsant treatment during pregnancy for school-age children and adolescents. Epilepsia, 1999. 40(9): p. 1237–43. 10487186 10.1111/j.1528-1157.1999.tb00852.x

[19] Speidel B.D. and Meadow S.R., Maternal epilepsy and abnormalities of the fetus and newborn. Lancet, 1972. 2(7782): p. 839–43. 4116552 10.1016/s0140-6736(72)92209-x

[20] Kaae J., , Epilepsy, anti-epileptic medication use and risk of cancer. Int J Cancer, 2014. 134(4): p. 932–8. 23901034 10.1002/ijc.28396

[21] Schüz J., Weihkopf T., & Kaatsch P, Medication use during pregnancy and the risk of childhood cancer in the offspring. European journal of pediatrics, 2007. 166(5): p. 433–441. 17345098 10.1007/s00431-006-0401-z

[22] Birch J.M., , The inter-regional epidemiological study of childhood cancer (IRESCC): case-control study of children with central nervous system tumours. Br J Neurosurg, 1990. 4(1): p. 17–25. 2334522 10.3109/02688699009000677

[23] La Vecchia C. and Negri E., A review of epidemiological data on epilepsy, phenobarbital, and risk of liver cancer. Eur J Cancer Prev, 2014. 23(1): p. 1–7. 23492954 10.1097/CEJ.0b013e32836014c8

[24] Dethloff L.A., , Perspective on the carcinogenic potential of phenytoin based on rodent tumor bioassays and human epidemiological data. Hum Exp Toxicol, 1996. 15(4): p. 335–48. 8845224 10.1177/096032719601500410

[25] Shaw A.K., Infante-Rivard C., and Morrison H.I., Use of medication during pregnancy and risk of childhood leukemia (Canada). Cancer Causes Control, 2004. 15(9): p. 931–7. 15577295 10.1007/s10552-004-2230-6

[26] Howe G.R., , An exploratory case-control study of brain tumors in children. Cancer Res, 1989. 49(15): p. 4349–52. 2743324

[27] Gurney J.G., , A study of pediatric brain tumors and their association with epilepsy and anticonvulsant use. Neuroepidemiology, 1997. 16(5): p. 248–55. 9346345 10.1159/000109694

[28] Gilman E.A., Wilson L. M., Kneale G. W., & Waterhouse J. A., Childhood cancers and their association with pregnancy drugs and illnesses. Paediatric and perinatal epidemiology, 1989. 3(1): p. 66–94. 2652101 10.1111/j.1365-3016.1989.tb00371.x

[29] Murray J.C., , Lymphoblastic lymphoma following prenatal exposure to phenytoin. J Pediatr Hematol Oncol, 1996. 18(2): p. 241–3. 8846150 10.1097/00043426-199605000-00033

[30] Sanders B.M. and Draper G.J., Childhood cancer and drugs in pregnancy. Br Med J, 1979. 1(6165): p. 717–8. 435748 10.1136/bmj.1.6165.717 PMC1598877

[31] Olsen J.H., Boice J.D. Jr., and Fraumeni J.F. Jr., Cancer in children of epileptic mothers and the possible relation to maternal anticonvulsant therapy. Br J Cancer, 1990. 62(6): p. 996–9. 2257233 10.1038/bjc.1990.424 PMC1971556

[32] Johnson K.J., , Childhood brain tumor epidemiology: a brain tumor epidemiology consortium review. Cancer Epidemiol Biomarkers Prev, 2014. 23(12): p. 2716–36. 25192704 10.1158/1055-9965.EPI-14-0207 PMC4257885

[33] Goldhaber M.K., , Exposure to barbiturates in utero and during childhood risk of intracranial and spinal cord tumors. Cancer Res, 1990. 50(15): p. 4600–3. 2369735

[34] Contreras Z.A., , Parental Age and Childhood Cancer Risk: A Danish Population-Based Registry Study. Cancer Epidemiology, 2017. 49: p. 202–215. 28715709 10.1016/j.canep.2017.06.010 PMC5586505

[35] Huff V., , Parental origin of de novo constitutional deletions of chromosomal band 11p13. Am J Hum Genet, 1990. 47(1): p. 155–60. 1971994 PMC1683741

[36] Szaflarski M., , Poverty, insurance, and region as predictors of epilepsy treatment among US adults. Epilepsy Behav, 2020. 107: p. 107050. 32294594 10.1016/j.yebeh.2020.107050 PMC7242147

[37] Ruda R., Trevisan E., and Soffietti R., Epilepsy and brain tumors. Curr Opin Oncol, 2010. 22(6): p. 611–20. 20706121 10.1097/CCO.0b013e32833de99d

[38] Li X., Sundquist J., and Sundquist K., Socioeconomic and occupational risk factors for epilepsy: a nationwide epidemiological study in Sweden. Seizure, 2008. 17(3): p. 254–60. 17728158 10.1016/j.seizure.2007.07.011 PMC2292825

[39] Ibrahim K. and Appleton R., Seizures as the presenting symptom of brain tumours in children. Seizure, 2004. 13(2): p. 108–12. 15129840 10.1016/s1059-1311(03)00083-9

[40] Maldonado G. and Greenland S., Simulation study of confounder-selection strategies. Am J Epidemiol, 1993. 138(11): p. 923–36. 8256780 10.1093/oxfordjournals.aje.a116813

[41] Gold E., , Increased risk of brain tumors in children exposed to barbiturates. J Natl Cancer Inst, 1978. 61(4): p. 1031–4. 279709

[42] Gloor P. and Fariello R.G., Generalized epilepsy: some of its cellular mechanisms differ from those of focal epilepsy. Trends Neurosci, 1988. 11(2): p. 63–8. 2465601 10.1016/0166-2236(88)90166-x

[43] Christensen J., , Incidence and prevalence of epilepsy in Denmark. Epilepsy Res, 2007. 76(1): p. 60–5. 17686613 10.1016/j.eplepsyres.2007.06.012

[44] Viale L., , Epilepsy in pregnancy and reproductive outcomes: a systematic review and meta-analysis. Lancet, 2015. 386(10006): p. 1845–52. 26318519 10.1016/S0140-6736(15)00045-8

[45] Seppala L.K., , Maternal Thyroid Disease and the Risk of Childhood Cancer in the Offspring. Cancers (Basel), 2021. 13(21). 10.3390/cancers13215409 PMC8582383 34771572

[46] Spector L.G., , Cancer risk among children with very low birth weights. Pediatrics, 2009. 124(1): p. 96–104. 19564288 10.1542/peds.2008-3069 PMC2704984

[47] Seppala L.K., , Preterm birth, neonatal therapies and the risk of childhood cancer. Int J Cancer, 2021. 148(9): p. 2139–2147. 33128776 10.1002/ijc.33376

[48] Shahla M., Hijran B., and Sharif M., The course of epilepsy and seizure control in pregnant women. Acta Neurol Belg, 2018. 118(3): p. 459–464. 29981006 10.1007/s13760-018-0974-0

[49] Bech B.H., , Use of antiepileptic drugs during pregnancy and risk of spontaneous abortion and stillbirth: population based cohort study. BMJ, 2014. 349: p. g5159. 25150301 10.1136/bmj.g5159 PMC4141333

[50] Werler M.M., , Use of antiepileptic medications in pregnancy in relation to risks of birth defects. Ann Epidemiol, 2011. 21(11): p. 842–50. 21982488 10.1016/j.annepidem.2011.08.002 PMC4816042

[51] Bertollini R., Mastroiacovo P., and Segni G., Maternal epilepsy and birth defects: a case-control study in the Italian Multicentric Registry of Birth Defects (IPIMC). Eur J Epidemiol, 1985. 1(1): p. 67–72. 3939491 10.1007/BF00162315

[52] Kelly T.E., , Teratogenicity of anticonvulsant drugs. II: A prospective study. Am J Med Genet, 1984. 19(3): p. 435–43. 6507489 10.1002/ajmg.1320190303

[53] Lupo P.J., , Association Between Birth Defects and Cancer Risk Among Children and Adolescents in a Population-Based Assessment of 10 Million Live Births. JAMA Oncol, 2019. 5(8): p. 1150–1158. 31219523 10.1001/jamaoncol.2019.1215 PMC6587148

[54] Heck J.E., , Spina bifida and pediatric cancers. Pediatr Hematol Oncol, 2020. 37(7): p. 630–636. 32364426 10.1080/08880018.2020.1760409 PMC7577564

[55] Coven S.L., , Delays in diagnosis for children with newly diagnosed central nervous system tumors. Neurooncol Pract, 2018. 5(4): p. 227–233. 31386013 10.1093/nop/npy002 PMC6655489

[56] Englot D.J., Chang E.F., and Vecht C.J., Epilepsy and brain tumors. Handb Clin Neurol, 2016. 134: p. 267–85. 26948360 10.1016/B978-0-12-802997-8.00016-5 PMC4803433

[57] Yasiry Z. and Shorvon S.D., How phenobarbital revolutionized epilepsy therapy: the story of phenobarbital therapy in epilepsy in the last 100 years. Epilepsia, 2012. 53 Suppl 8: p. 26–39. 10.1111/epi.12026 23205960

[58] Ernst L.L., , Medication adherence in women with epilepsy who are planning pregnancy. Epilepsia, 2016. 57(12): p. 2039–2044. 27778312 10.1111/epi.13586 PMC6374285

[59] Cohen J.M., , Prevalence trends and individual patterns of antiepileptic drug use in pregnancy 2006-2016: A study in the five Nordic countries, United States, and Australia. Pharmacoepidemiol Drug Saf, 2020. 29(8): p. 913–922. 32492755 10.1002/pds.5035

[60] Singh G., Driever P.H., and Sander J.W., Cancer risk in people with epilepsy: the role of antiepileptic drugs. Brain, 2005. 128(1): p. 7–17. 15574465 10.1093/brain/awh363

[61] Brodie M.J. and Kwan P., Current position of phenobarbital in epilepsy and its future. Epilepsia, 2012. 53: p. 40–46. 23205961 10.1111/epi.12027

